# Loss of SFXN1 mitigates lipotoxicity and predicts poor outcome in non-viral hepatocellular carcinoma

**DOI:** 10.1038/s41598-023-36660-w

**Published:** 2023-06-09

**Authors:** Kohei Yagi, Shu Shimada, Yoshimitsu Akiyama, Megumi Hatano, Daisuke Asano, Yoshiya Ishikawa, Hiroki Ueda, Shuichi Watanabe, Keiichi Akahoshi, Hiroaki Ono, Minoru Tanabe, Shinji Tanaka

**Affiliations:** 1 Department of Molecular Oncology, Graduate School of Medicine, Tokyo Medical and Dental University, 1-5-45 Yushima, Bunkyo-Ku, Tokyo, 113-8519 Japan; 2 Department of Hepato-Biliary-Pancreatic Surgery, Graduate School of Medicine, Tokyo Medical and Dental University, Tokyo, Japan

**Keywords:** Hepatology, Surgical oncology

## Abstract

Hepatocellular carcinoma (HCC) imposes a huge global burden, arising from various etiological factors such as hepatitis virus infection and metabolic syndrome. While prophylactic vaccination and antiviral treatment have decreased the incidence of viral HCC, the growing prevalence of metabolic syndrome has led to an increase in non-viral HCC. To identify genes downregulated and specifically associated with unfavorable outcome in non-viral HCC cases, screening analysis was conducted using publically available transcriptome data. Among top 500 genes meeting the criteria, which were involved in lipid metabolism and mitochondrial function, a serine transporter located on inner mitochondrial membrane SFXN1 was highlighted. SFXN1 protein expression was significantly reduced in 33 of 105 HCC tissue samples, and correlated to recurrence-free and overall survival only in non-viral HCC. Human HCC cells with *SFXN1* knockout (KO) displayed higher cell viability, lower fat intake and diminished reactive oxygen species (ROS) production in response to palmitate administration. In a subcutaneous transplantation mouse model, high-fat diet feeding attenuated tumorigenic potential in the control cells, but not in the SFXN1-KO cells. In summary, loss of SFXN1 expression suppresses lipid accumulation and ROS generation, preventing toxic effects from fat overload in non-viral HCC, and predicts clinical outcome of non-viral HCC patients.

## Introduction

Liver cancer is a global health issue of great concern, ranking sixth in morbidity and second in mortality and being the second leading cause of years of life lost to cancer^1,2^. Hepatocellular carcinoma (HCC) accounts for 70 to 85% of primary liver cancer cases^1^, and is linked to a variety of risk factors including chronic infection with hepatitis B virus (HBV) or hepatitis C virus (HCV), alcohol abuse and metabolic disease^3^. Universal HBV vaccination programs have dramatically diminished HBV infection and HCC incidence^4^, and pharmacological suppression of HBV and clearance of HCV decrease HCC occurrence by 50 to 80%^5^. On the other hand, non-alcoholic fatty liver disease (NAFLD) and non-alcoholic steatohepatitis (NASH), associated with metabolic syndrome including obesity and type 2 diabetes, have become the most common liver diseases and major drivers of HCC in developed countries^5^. Although patients with NASH have a lower annual incidence of HCC (1 to 2%) compared to those with HCV-related cirrhosis, the rising prevalence of NASH is now shifting the etiology of HCC from viral infection to metabolic disease^3^.

To improve patient outcome, it is essential to categorize HCC samples into subtypes, understand molecular mechanisms of each subtype and develop subtype-specific therapies. A two-group model for HCC classification based on gene expression patterns, the aggressive and non-aggressive groups, is widely accepted in recent years^6,7^. Our latest integrated analysis of genome, transcriptome and clinicopathological data has revealed that the non-aggressive group is further divided into the catenin beta-1 (*CTNNB1*)-mutated subtype and the metabolic disease-associated subtype, the latter of which is mainly composed of non-viral HCC^8^. However, no specific genetic alterations are identified in the non-viral/metabolic disease-associated subtype, suggesting that gene expression changes may contribute to hepatocarcinogenesis in this subtype.

In this study, we first screened genes involved in clinical outcome of non-viral HCC patients using publically available data from the Cancer Genome Atlas Research Network (TCGA), which contained gene expression profiles and clinical information on viral infection, but not metabolic syndrome. Gene ontology analysis demonstrated the downregulation of genes associated with lipid metabolism and mitochondrial function in non-viral HCC. We investigated the most promising candidate gene *SFXN1*, a serine transporter located on inner mitochondrial membrane, among them.

## Results

### Identification of genes defining patient prognosis in non-viral HCC

**Figure 1:**
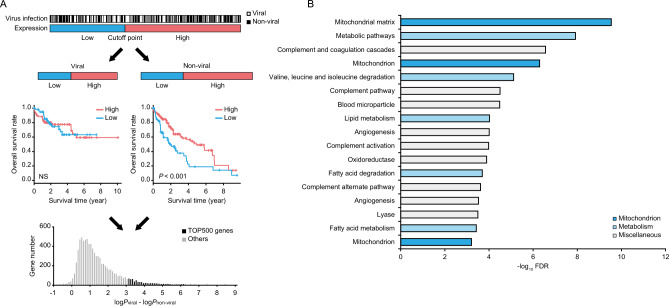
Identification of genes defining patient prognosis in non-viral HCC. (**A**) Screening analysis of the TCGA data for detecting genes downregulated and specifically associated with poor prognosis in non-viral HCC. Of 8,190 genes satisfying the criteria, 500 genes with the highest difference of *P*-values between the viral and non-viral HCC groups (Δlog*P* = log*P*_viral_ − log*P*_non-viral_) are designated as “TOP500” genes. Details are described in the Methods and Results sections. (**B**) DAVID gene ontology analysis of the TOP500 genes. All of the 17 annotations with a FDR < 0.001 are shown. *NS*: not significant; *FDR*: false discovery rate.

We analyzed a total of 20,531 genes in 370 HCC patients, comprising 153 viral and 217 non-viral cases, using the TCGA datasets. While determining the optimal cutoff points (Fig. 1A, upper panel), we compared overall survival between the subgroups of patients with the high and low expression levels in each of viral and non-viral HCC groups for each gene (Fig. 1A, middle panel), and calculated the difference of *P*-values between the viral and non-viral HCC groups (Δlog*P* = log*P*_viral_ − log*P*_non-viral_). Of 8,190 genes meeting the criterion that overall survival was worse in the low expression subgroup than in the high expression subgroup in the non-viral HCC group, we selected 500 genes with the highest Δlog*P*, designated as “TOP500” genes (Fig. 1A, lower panel; Supplementary Table 1).

Gene ontology analysis of the TOP500 genes with the DAVID program^9^ identified 17 annotations with a false discovery rate < 0.001 (Supplementary Table 2). These annotations included five related to metabolism, particularly three related to lipid metabolism (Fig. 1B), consistent with previous studies on the close relationship between non-viral HCC and metabolic disorder^5–8^. Notably, the 17 annotations also contained three related to mitochondria (Fig. 1B), suggesting a potential role of mitochondrial dysfunction in non-viral hepatocarcinogenesis. Therefore, we highlighted *SFXN1*, which had the highest Δlog*P* among genes commonly included in three inner mitochondrial membrane-related gene sets of the MSigDB collection (Supplementary Table 3–5).

### Evaluation of SFXN1 expression and patient survival in HCC tissues

**Figure 2:**
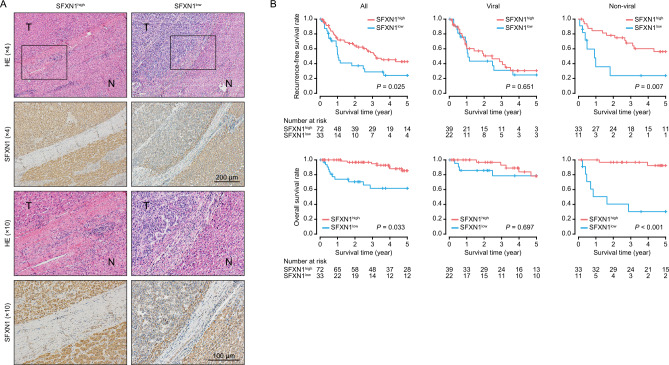
Evaluation of SFXN1 expression and patient survival in HCC tissues. (**A**) Representative immunohistochemical images of SFXN1 in HCC specimens. N: adjacent liver tissue; T: tumor tissue; HE: hematoxylin and eosin staining. (**B**) Kaplan–Meier curves of recurrence-free (upper) and overall (lower) survival in all HCC patients (left; n = 105), viral (middle; n = 61) and non-viral HCC (right; n = 44) groups. *P*-values are calculated by the log-rank test.

Immunohistochemical staining was performed to assess SFXN1 protein expression in 105 HCC samples surgically resected in our institution, which were classified into the high and low expression groups (*n* = 72 and 33, respectively) based on the intensity score of SFXN1 staining (Fig. 2A). In both groups, the protein expression level of SFXN1 was downregulated in tumor tissues compared to adjacent liver tissues, with a more pronounced decrease observed in the low expression group (Supplementary Fig. 1). Although there was no obvious connection between SFXN1 expression and clinicopathological factors (Tables 1, 2, 3), the low SFXN1 expression group demonstrated poorer recurrence-free survival (*P* = 0.025) and overall survival (*P* = 0.033) compared to the high SFXN1 expression group (Fig. 2B). Further subanalysis revealed that low SFXN1 expression was strongly associated with unfavorable recurrence-free survival (*P* = 0.007) and overall survival (*P* < 0.001) in the non-viral HCC group, but not in the viral HCC group. These findings suggest that SFXN1 expression may serve as a specific prognostic factor for non-viral HCC, and confirm the results obtained from screening analysis of the TCGA data (Fig. 1).

**Table 1:** Relationship between SFXN1downregulation and clinicopathological factors.

Variable	SFXN1^high^ (*n* = 72)	SFXN1^low^ (*n* = 33)	*P*-value
Age (median)	72 (48–85)	74 (37–86)	0.51
Sex			
Male	54	28	0.32
Female	18	5	
Viral infection			
Negative	33	11	0.23
Positive	39	22	
HBV	7	7	0.10
HCV	32	15	0.92
Matteoni			
0	18	6	0.87
1, 2	8	2	
3, 4	7	3	
Alcohol	37	20	0.38
Diabetes	23	13	0.45
Dyslipidemia	11	9	0.35
Obesity			
BMI > 25	28	15	0.53
BMI ≤ 25	44	18	
Plt			
< 10 × 10^4^/μL	8	4	1.00
≥ 10 × 10^4^/μL	64	29	
PT			
< 80%	12	6	1.00
≥ 80%	60	27	
Alb			
< 3.5 g/dL	11	3	0.54
≥ 3.5 g/dL	61	30	
AST			
> 40 IU/L	32	12	0.44
≤ 40 IU/L	40	21	
ALT			
> 40 IU/L	27	8	0.26
≤ 40 IU/L	45	25	
T-Bil			
> 1.0 mg/dL	13	8	0.60
≤ 1.0 mg/dL	59	25	
ICG			
> 15%	28	14	0.73
≤ 15%	44	19	
α-Fetoprotein (AFP)			
> 200 ng/mL	16	10	0.37
≤ 200 ng/mL	56	23	
PIVKA-II			
> 100 mAU/mL	35	17	0.78
≤ 100 mAU/mL	37	15	
Child–Pugh grade			
A	71	32	0.53
B	1	1	
Tumor size			
> 5 cm	23	11	0.89
≤ 5 cm	49	22	
Tumor number			
Solitary	60	24	0.21
Multiple	12	9	
Differentiation			
Well	27	9	0.31
Poorly/moderately	45	24	
Portal vein invasion			
0	51	19	0.18
1, 2	19	14	
3, 4	2	0	
Liver status			
NL	15	5	0.60
CH/LC	57	28	

*BMI* body mass index, *NL* normal liver, *CH* chronic hepatitis, *LC* liver cirrhosis.

**Table 2:** Relationship between SFXN1downregulation and clinicopathological factors in viral cases.

Variable	SFXN1^high^ (*n* = 39)	SFXN1^low^ (*n* = 22)	*P*-value
Age (median)	70 (49–82)	74 (37–86)	0.18
Sex			
Male	27	17	0.50
Female	12	5	
Viral infection			
HBV	7	7	0.21
HCV	32	15	
Alcohol	17	13	0.24
Diabetes	8	8	0.18
Dyslipidemia	2	4	0.23
Obesity			
BMI > 25	13	7	0.90
BMI ≤ 25	26	15	
Plt			
< 10 × 10^4^/μL	6	4	0.78
≥ 10 × 10^4^/μL	33	18	
PT			
< 80%	6	4	0.78
≥ 80%	33	18	
Alb			
< 3.5 g/dL	7	2	0.57
≥ 3.5 g/dL	32	20	
AST			
> 40 IU/L	23	6	0.017*
≤ 40 IU/L	16	16	
ALT			
> 40 IU/L	17	4	0.045*
≤ 40 IU/L	22	18	
T-Bil			
> 1.0 mg/dL	9	6	0.71
≤ 1.0 mg/dL	30	16	
ICG			
> 15%	18	10	0.96
≤ 15%	21	12	
α-Fetoprotein (AFP)			
> 200 ng/mL	10	8	0.38
≤ 200 ng/mL	29	14	
PIVKA-II			
> 100 mAU/mL	26	13	0.55
≤ 100 mAU/mL	13	9	
Child–Pugh grade			
A	39	22	1.00
B	0	0	
Tumor size			
> 5 cm	9	5	0.98
≤ 5 cm	30	17	
Tumor number			
Solitary	30	17	0.98
Multiple	9	5	
Differentiation			
Well	15	6	0.37
Poorly/moderately	24	16	
Portal vein invasion			
0	26	12	0.16
1, 2	10	10	
3, 4	3	0	
Liver status			
NL	2	2	0.95
CH/LC	37	20	

*BMI* body mass index, *NL* normal liver, *CH* chronic hepatitis, *LC* liver cirrhosis.

**Table 3:** Relationship between SFXN1downregulation and clinicopathological factors in non-viral cases.

Variable	SFXN1^high^ (*n* = 33)	SFXN1^low^ (*n* = 11)	*P*-value
Age (median)	74 (48–85)	73 (62–82)	0.66
Sex			
Male	27	11	0.12
Female	6	0	
Matteoni			
0	18	6	0.87
1, 2	8	2	
3, 4	7	3	
Alcohol	20	7	0.88
Diabetes	15	5	1.00
Dyslipidemia	9	5	0.26
Obesity			
BMI > 25	15	8	0.12
BMI ≤ 25	17	3	
Plt			
< 10 × 10^4^/μL	2	0	0.40
≥ 10 × 10^4^/μL	31	11	
PT			
< 80%	6	2	1.00
≥ 80%	27	9	
Alb			
< 3.5 g/dL	4	1	0.78
≥ 3.5 g/dL	29	10	
AST			
> 40 IU/L	9	6	0.10
≤ 40 IU/L	24	5	
ALT			
> 40 IU/L	10	4	0.71
≤ 40 IU/L	23	7	
T-Bil			
> 1.0 mg/dL	4	2	0.61
≤ 1.0 mg/dL	29	9	
ICG			
> 15%	10	4	0.71
≤ 15%	23	7	
α-Fetoprotein (AFP)			
> 200 ng/mL	6	2	1.00
≤ 200 ng/mL	27	9	
PIVKA-II			
> 100 mAU/mL	14	6	0.48
≤ 100 mAU/mL	19	5	
Child–Pugh grade			
A	33	10	0.56
B	0	1	
Tumor size			
> 5 cm	14	6	0.48
≤ 5 cm	19	5	
Tumor number			
Solitary	30	7	0.10
Multiple	3	4	
Differentiation			
Well	12	3	0.58
Poorly/moderately	21	8	
Portal vein invasion			
0	25	6	0.18
1, 2	8	5	
3, 4	0	0	
Liver status			
NL	13	3	0.47
CH/LC	20	8	

*BMI* body mass index, *NL* normal liver, *CH* chronic hepatitis, *LC* liver cirrhosis.

### Evaluation of biological effects of *SFXN1* knockout on HCC cells

**Figure 3:**
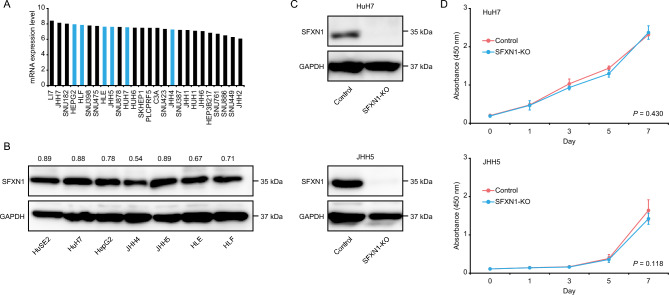
Establishment of the SFXN1-KO HCC cells. (**A**) mRNA expression analysis of *SFXN1* in 26 human HCC cell lines using the Cancer Cell Line Encyclopedia datasets. (**B**) Protein expression analysis of SFXN1 in immortalized hepatocytes HuSE2 and six human liver cancer cell lines. The SFXN1/GAPDH protein expression ratios are displayed above the blot images. (**C**) Immunoblot analysis of SFXN1 in the SFXN1-KO HCC cells. (**D**) Cell proliferation assay of the SFXN1-KO HCC cells. *P*-values are calculated by Welch’s *t*-test. Data are the mean ± SD. NS: not significant.

We next assessed the expression level of *SFXN1* in 26 human HCC cells using publicly available transcriptome data from the Cancer Cell Line Encyclopedia and conducting Western blot analysis of immortalized but not transformed human hepatocytes HuSE2 and six human liver cancer cell lines. SFXN1 was generally expressed in HCC cell lines at both the mRNA and protein levels (Fig. 3A, B), and the protein expression level of SFXN1 was downregulated in human liver cancer cell lines compared to human hepatocytes (Fig. 3B). The JHH4 cells exhibited the relatively low mRNA and protein expression levels of *SFXN1*, suggesting a positive correlation between mRNA and protein expression of *SFXN1*. Because the HuH7 and JHH5 cells displayed the high SFXN1 expression level among liver cancer cell lines, *SFXN1* knockout (SFXN1-KO) was generated in the two HCC cell lines using the clustered regularly interspaced short palindromic repeats (CRISPR)/Cas9 system (Fig. 3C). There was no critical effect of SFXN1-KO on the proliferative activity of these cells (Fig. 3D).

**Figure 4:**
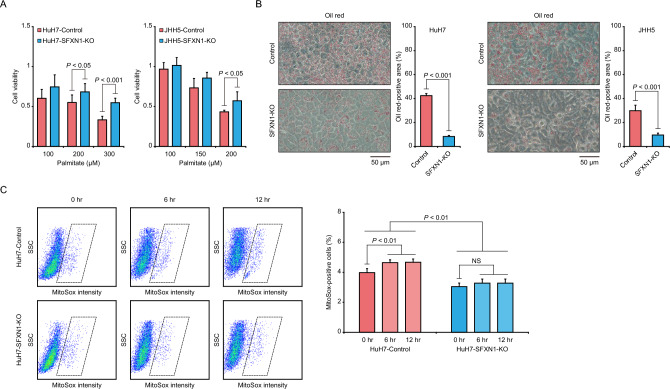
Evaluation of biological effects of SFXN1-KO on HCC cells. (**A**) Cell viability assay of the SFXN1-KO HCC cells treated with palmitate. *P*-values are calculated by Welch’s *t*-test. Data are the mean ± SD. (**B**) Oil red staining of the SFXN1-KO HCC cells treated with palmitate. The left and right panels show representative images and quantification data of cells stained with oil red, respectively. *P*-values are calculated by Welch’s *t*-test. Data are the mean ± SD. (**C**) Mitochondrial ROS production in response to palmitate administration in the SFXN1-KO HCC cells. The left and right panels show representative flow cytometry plots and quantitative data of the percentage of MitoSox-positive cells, respectively. *P*-values are calculated by ANOVA with Tukey–Kramer post hoc test. Data are the mean ± SD. SSC: side scatter; NS: not significant.

Based on our findings (Fig. 1B), we hypothesized that SFXN1 might play an important role in lipid metabolism and mitochondrial function in HCC, particularly under abnormal metabolic conditions. To test the hypothesis, we exposed the SFXN1-KO and control subclones derived from the HuH7 and JHH5 cells to palmitate, and observed that the cell viability of the SFXN1-KO cells was higher than that of the control cells (Fig. 4A). Oil red staining following palmitate administration revealed less fat intake in the SFXN1-KO cells than in the control cells (Fig. 4B). Additionally, we evaluated mitochondrial reactive oxygen species (ROS) production in response to palmitate loading using the MitoSOX mitochondrial superoxide indicator. The HuH7-Control cells showed the higher ROS level than the HuH7-SFXN1-KO cells, and palmitate treatment increased ROS production in the HuH7-Control cells, but not in the HuH7-SFXN1-KO cells (Fig. 4C). Taken together, SFXN1 attenuation confers resistance to lipotoxicity through the reduction of mitochondrial ROS generation.

### Evaluation of in vivo effects of SFXN1 attenuation on lipotoxicity

**Figure 5:**
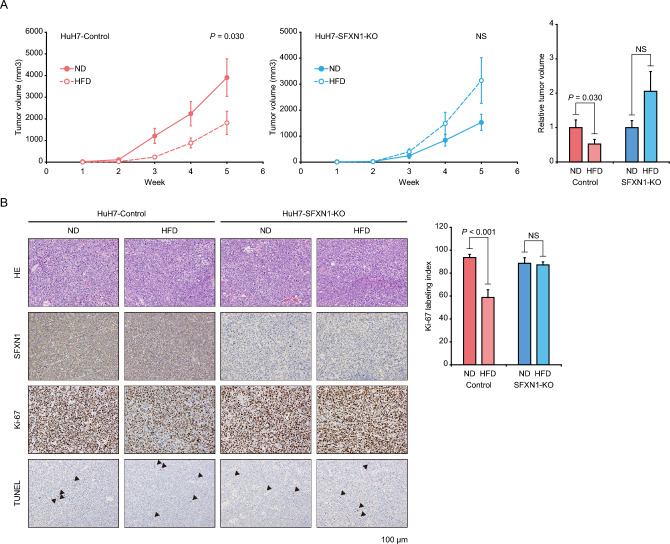
Evaluation of in vivo effects of SFXN1 attenuation on lipotoxicity. (**A**) Tumorigenicity assay of the SFXN1-KO HCC cells in immunodeficient mice fed with HFD. The left and right panels show tumor growth curves and relative tumor volumes at five weeks. *P*-values are calculated by Welch’s *t*-test. Data are the mean ± SE. (**B**) Immunohistochemical analysis of tumor tissues derived from the SFXN1-KO HCC cells in immunodeficient mice with HFD. The left and right panels show representative immunohistochemical images and quantitative data of the percentage of Ki-67-positive cells (Ki-67 labeling index), respectively. Arrowheads indicate TUNEL-positive cells. ND: normal diet; HFD: high-fat diet; NS: not significant.

In order to examine in vivo effects of SFXN1-KO on lipotoxicity, we subcutaneously injected the HuH7-Control and HuH7-SFXN1-KO cells into nude mice, and fed the tumor-bearing mice with either normal diet (ND) or high-fat diet (HFD). Treatment with HFD significantly suppressed tumor growth of the HuH7-Control cells compared to treatment with ND, while there was no difference in tumor development of the HuH7-SFXN1-KO cells between ND and HFD administration (Fig. 5A). There was a tendency that the tumor size of the HuH7-SFXN1-KO cells was larger than that of the HuH7-Control cells under HFD feeding, but no significant difference was observed (Supplementary Fig. 2). Immunohistochemical analysis of the grafted tumors confirmed a decrease in SFXN1 protein expression in the SFXN1-KO tumor tissues (Fig. 5B), and revealed that the Ki-67 labeling index was markedly lower in tumor specimens of the Control-HFD group in comparison to the other three groups (Fig. 5B). Cleaved caspase-3-positive and TUNEL-positive cells were rarely observed in all the four groups (Fig. 5B and Supplementary Fig. 3).

## Discussion

*Sfxn1* (sideroflexin-1) was originally identified as the gene mutated in a mouse mutant with anemia and axial skeletal abnormalities, however, its molecular roles remained unclear. Kory et al*.* have revealed that SFXN1 transports serine on the inner mitochondrial membrane, and functions as an essential component of the serine one-carbon metabolism pathway^10^. SFXN1 is highly expressed in tissues with high one-carbon metabolism activity, such as blood and liver. Zhang et al*.* have discovered that the liver drives acetyl-CoA for lipogenesis from acetate and lactate and nicotinamide adenine dinucleotide phosphate (NADPH) from folate-mediated one-carbon metabolism, while de novo lipogenesis in adipose tissues is supported by glucose and its catabolism via the pentose phosphate pathway to produce NADPH^11^. In fact, inhibition of the one-carbon metabolism pathway reduces hepatic lipogenesis and steatosis in mice. Besides one-carbon metabolism, other roles of SFXN1 in mitochondrial function have recently been reported; loss of SFXN1 compromises mitochondrial complex III and impairs respiratory chain activity, and SFXN1 is important for coenzyme Q, heme and α-ketoglutarate metabolism^12^; enhanced protein expression of SFXN1 contributes to an increase in iron absorption, which causes sideroblastic anemia, and SFXN1 delivers cytoplasmic Fe^2+^ into the mitochondria, giving rise to mitochondrial ROS synthesis and ferroptosis^13^. The relationship between SFXN1 expression, lipid accumulation and ROS generation observed in the previous studies is consistent with our results (Fig. 4).

Screening analysis of the TCGA data identified that genes involved in patient prognosis were associated with lipid metabolism and mitochondrial function. The liver is a key organ for the metabolism of glucose, fatty acids and amino acids in the mitochondria, and mitochondrial dysfunction is one of critical factors in the pathogenesis of non-viral HCC through various biological processes including ROS production. We have previously reported two papers on the relationship between ROS and HCC. One is that cytochrome P450 1A2 (CYP1A2) downregulation predicts recurrence of HCC and is associated with the oxidative stress pathway^14^, and the other is that cancer stem cells of HCC are characterized by the low proteasome activity and the low intracellular ROS level^15^. Mitochondrial ROS generation plays both oncogenic and tumor-suppressive roles in tumorigenesis. At low to moderate levels, ROS stimulates mitogen-activated protein kinase (MAPK) phosphorylation, cyclin D1 expression and c-Jun N-terminal kinase (JNK) activation, all of which are linked to cancer cell growth and survival^16^, and also induces mutagenesis by formation of 8-oxoguanine, which can lead to G-T or G-A transversion^17^. On the other hand, excess cellular levels of ROS damage proteins, lipids, nucleic acids and organelles, which can trigger cell death cascades. Mitochondrial ROS can initiate intrinsic apoptosis by releasing cytochrome *c* into the cytosol, and transmembrane death receptors like Fas and TNF-related apoptosis-inducing ligand receptor (TRAIL-R)1/2 can be activated by ROS as the extrinsic apoptosis pathway^18^. An increase in ROS production enhances lipid peroxidation, thereby inducing ferroptosis, a recently discovered type of Fe^2+^-dependent programmed cell death^19^. Since SFXN1 is implicated not only in ROS generation but also in iron metabolism, including increased iron absorption and Fe^2+^ transport from the cytoplasm into the mitochondria^13^, SFXN1 downregulation may potentially inhibit the ferroptotic pathway and confer resistance to lipotoxicity. Further analysis is required to elucidate the detailed molecular mechanism underlying the role of SFXN1 in lipotoxicity.

Cells of non-adipose tissues are able to store lipids to a limited extent, which induces ROS synthesis, endoplasmic reticulum stress and inflammatory response. Endoplasmic reticulum stress and fat overload cooperatively lead to NASH and steatohepatitic HCC development by attracting and activating inflammatory macrophages that secret tumor necrosis factors^20^. Free fatty acids can control mitochondrial ROS generation by several mechanism: (1) Interference with the electron transport. (2) Impairment of the glutathione system. (3) Modulation of mitochondrial membrane fluidity^21^. In fact, when hepatocytes and HCC cells are exposed to high free fatty acid concentrations, accelerated mitochondrial fatty acid β-oxidation causes excessive electron flux in the electron transport chain and ROS overproduction, resulting in lipotoxicity^22^. Thus, lipid accumulation exerts positive and negative effects on hepatocarcinogenesis. This study shows that high-fat diet feeding reduces tumor growth of HCC cells (Fig. 5A), suggesting the latter mechanism, and that *SFXN1* knockout attenuates lipid accumulation in vitro (Fig. 4B) and mitigates lipotoxicity in vivo (Fig. 5A).

In summary, our bioinformatics and immunohistochemical analysis demonstrate a close correlation between SFXN1 downregulation and poor outcome in non-viral HCC. Further investigation in human HCC cells and in a xenograft mouse model validates the roles of SFXN1 in lipid metabolism and mitochondrial function, and clarifies that loss of SFXN1 expression facilitates tumorigenic property under fat-loading conditions in non-viral HCC. These results shed light on the significance of SFXN1 in the pathogenesis of non-viral HCC, and suggest SFXN1 as not only a putative prognostic biomarker but also a potential therapeutic target for the management of this subtype.

## Materials and methods

### Ethics statement

All experimental protocols were approved by Institutional Review Board (G2017-018, Medical Research Ethics Committee for Life Science of Tokyo Medical and Dental University; G2018-132C10, Medical Research Ethics Committee for Genetic Research of Tokyo Medical and Dental University; A2021-059C2, Institutional Animal Care and Use Committee of Tokyo Medical and Dental University). The study involving humans reports to be in accordance with relevant guidelines and regulations. All methods on animals were carried out in accordance with Guidelines for Proper Conduct of Animal Experiments of Science Council of Japan (https://www.scj.go.jp/en/animal/). This study was carried out in compliance with the ARRIVE guidelines (https://arriveguidelines.org/arrive-guidelines).

### Bioinformatics analysis

Transcriptome and clinical data of 370 HCC patients, including 153 viral and 217 non-viral cases, were provided from TCGA (https://www.cancer.gov/ccg/research/genome-sequencing/tcga), and downloaded from the cBioPortal site (https://www.cbioportal.org/). For each of 20,531 genes, Δlog*P* was determined as follows (Fig. 1A); (1) expression labels of “high” (*n* cases) and “low” (370 − *n* cases) were assigned to the HCC patients on the basis of their gene expression levels, (2) overall survival was compared between the high and low expression subgroups in each of the viral and non-viral HCC groups using the log-rank test, (3) the maximum difference of *P*-values between the viral and non-viral HCC groups (Δlog*P* = log*P*_viral_ − log*P*_non-viral_) was computed by varying *n* from the lower quartile to the upper quartile. Gene ontology analysis was performed using the Database for Annotation, Visualization and Integrated Discovery (DAVID) program according to the developer’s instruction^9^. Transcriptome data of 26 HCC cell lines were provided from the Cancer Cell Line Encyclopedia (https://sites.broadinstitute.org/ccle/), and downloaded from the DepMap site (https://depmap.org/portal/).

### Human tissue samples

A total of 105 patients, including 61 viral HCC and 44 non-viral HCC cases, underwent curative resection for HCC at Tokyo Medical and Dental University Hospital between 2013 and 2016. All the patients provided informed consent before enrollment and were anonymously coded in accordance with ethical guidelines.

### Immunohistochemical analysis

Tissues were fixed overnight in Mildform 20N (Wako, Osaka, Japan), embedded in paraffin, and sectioned at 4 μm thickness. Sections were immersed in sodium citrate (pH 6.0) buffer for antigen retrieval, and subsequently incubated at 4 °C overnight with primary antibodies as follows; SFXN1 (12296-1-AP, 1:400; ProteinTech, Rosemont, IL), Ki-67 (D3B5, 1:400; Cell Signaling Technology, Danvers, MA) and cleaved caspase-3 (5A1E, 1:2000; Cell Signaling Technology). They were probed with anti-rabbit IgG antibody labelled with Histofine Simple Stain MAX-PO (Nichirei Bioscience, Tokyo, Japan) and visualized with diaminobenzidine (Wako). The TUNEL assay was conducted using DeadEnd Colorimetric TUNEL System (Promega, Madison, WI) according to the manufacture’s protocol. Nuclei were stained with hematoxylin. The intensity score of SFXN1 staining was determined in HCC tissues and adjacent liver tissues of each sample, ranging from 0 to 4. Tumor samples with a score of 0 or 1 and with a score of 2, 3 or 4 were categorized into the low and high expression groups, respectively.

### Cell culture

Human liver cancer cell lines HuH7, HepG2, JHH4, JHH5, HLE and HLF were purchased from the American Type Culture Collection (Manassas, VA) and the Human Science Research Resources Bank (Osaka, Japan), respectively. Cells were cultured in DMEM (Wako) or RPMI-1640 (Wako) medium containing 10% fetal bovine serum, and 1% penicillin, streptomycin and amphotericin B (Wako), maintained in a humidified incubator at 37 °C in 5% CO2, and harvested with 0.05% trypsin-0.03% EDTA (Wako). The HuSE2 cells are immortalized human hepatocytes with transduction of hTERT and HPV-E6/E7 oncogenes^23^, which were established by Dr. Kunitada Shimotohno’s group and provided from Dr. Takaji Wakita and Dr. Makoto Hijikata. The culture conditions of the HuSE2 cells were previously described^23^.

### Genome engineering

A CRISPR-target sequence for *SFXN1* knockout is 5′-TGGTTAACAGAATGTTCCTG-3′. Oligos were cloned into the lentiGuide-Puro vector (#52,963; Addgene, Watertown, MA) according to the developer’s instruction. The lentiCas9-Blast vector (#52,962; Addgene) was used for constitutively expressing *Sp*Cas9. The HEK293T cells were transfected with the lentiviral transfer plasmids, pCMVΔR8.2 and pHCMV-VSV-G using polyethylenimine (Polysciences, Warrington, PA). Culture supernatants were collected and passed through 0.45 μm-membrane filters (Merck Millipore, Burlington MA) two to three days after the transfection. Cells were infected for 12 h in the supernatant, and then treated with antibiotics such as 10 μg/mL puromycin (Thermo Fisher Scientific, Waltham, MA) and 10 μg/mL blasticidin S (Wako) for two days. Cell pellets were suspended in TNE buffer (10 mM Tris–HCl, pH 8.0; 150 mM NaCl; 2 mM EDTA; 0.5% SDS) with 1% proteinase K (TaKaRa Bio, Shiga, Japan) at 55 °C overnight, and genomic DNA was obtained by phenol–chloroform extraction. After genomic DNA was amplified with a primer set (forward, 5′-TTAGCTTTGAATTTAGATGGCC-3′; reverse, 5′-TGAAGGTTTAAAGATAGCAGCC-3′) at the *SFXN1* locus, PCR products were purified using QIAquick PCR Purification Kit (QIAGEN, Hulsterweg, Germany) and directly sequenced for checking gene knockout by Azenta Japan (Tokyo, Japan).

### Western blot analysis

After whole cell lysates were collected using ice-cold RIPA buffer (Thermo Fisher Scientific), 30 μg of protein from each sample was subjected to electrophoresis through 10% sodium dodecyl sulfate–polyacrylamide gels and transferred onto Immobilon polyvinyldifluoride membranes (Merck Millipore). The membrane was blocked with 5% skimmed milk or bovine serum albumin for an hour at room temperature, and then incubated overnight at 4 °C with primary antibodies as follows; SFXN1 (12296-1-AP, 1:1000; ProteinTech) and glyceraldehyde-3-phosphate dehydrogenase (GAPDH) (14C10, 1:2000; Cell Signaling Technology). Secondary antibodies were added, and signals were detected using Clarity Western ECL Substrate (Bio-Rad, Hercules, CA) with LAS-4000 mini (Fujifilm, Tokyo, Japan). GAPDH was used as an internal control. The protein expression levels of SFXN1 and GAPDH were semi-quantified using ImageJ (National Institutes of Health, Bethesda, MD).

### Cell proliferation assay

Cells were seeded at a density of 1 × 10^3^ cells per well in 96-well plates, and incubated overnight before each assay. To quantify the number of cells, 10 μL of WST-8 (Cell Counting Kit-8; Dojindo, Kumamoto, Japan) per well was added, and the absorbance was measured on a microplate reader (Bio-Rad Laboratories) at 450 nm with background subtraction at 630 nm at four hours. All experiments were done in quadruplicate.

### Cell viability assay

Cells were seeded in 100 μL of culture medium including 5 × 10^3^ cells into 96-well plates. Three days after cells were treated with various doses of palmitate solution, the number of live cells was measured using WST-8 as described above. Palmitate solution at a concentration of 20 mM was prepared as previously described^24^. Briefly, 10 μl of palmitate (Sigma Aldrich, St. Louis, MO) was preheated at 37 °C and mixed with 90 μl of bovine serum albumin by pipetting, followed by the addition of 150 μl of medium. The process of pipetting and incubation at 37 °C was repeated until the lipid particles became invisible. The mixture of 10 μl of ethanol, 90 μl of bovine serum albumin and 150 μl of medium was used as a vehicle. All experiments were done in quadruplicate.

### Oil red staining

Cells were seeded at a density of 5 × 10^5^ cells per well in 6-cm dishes, and exposed to culture medium containing 100 μM of palmitate solution for 24 h. Cells were washed with phosphate-buffered saline, fixed with 4% paraformaldehyde and stained with Oil red O solution (Merck Millipore). Stained cells were quantified using ImageJ. All experiments were done in duplicate.

### Mitochondrial ROS detection

Cells were seeded in 6-cm dishes, and exposed to culture medium containing 100 μM of palmitate solution for 24 h. Cell pellets were incubated in 5 μM of the MitoSox Green Reagent (Thermo Fisher Scientific) for 15 min at 37 °C, and subsequently washed with phosphate-buffered saline. The fluorescence intensity produced by the MitoSox Green Reagent was analyzed using FACSCalibur (BD Biosciences, San Jose, CA). The percentage of MitoSox-positive cells was calculated using WinMDI 2.8. All experiments were done in triplicate.

### Tumor seeding and HFD treatment

After suspended in 100 μL Matrigel (BD Biosciences), 1 × 10^6^ cells were subcutaneously inoculated into six-week-old male KSN nude mice (*n* = 4), which were purchased from Sankyo Labo Service Corporation (Tokyo, Japan). Tumor-bearing mice were fed with either normal diet (CE-2; CLEA Japan, Tokyo, Japan) or high-fat diet (HFD-60; Oriental Yeast, Tokyo, Japan). Mice were maintained in a temperature-controlled room (22–25 °C) on a 12-h light/12-h dark cycle with free access to food and water.

### Statistical analysis

All statistical analysis was performed with EZR (Saitama Medical Center, Jichi Medical University, Saitama, Japan), which is a graphical user interface for R (The R Foundation for Statistical Computing, Vienna, Austria). The two-sample comparison test was evaluated by Welch’s *t*-test after the normal distribution of the data was validated by the Kolmogorov–Smirnov test. The multiple comparison test was evaluated by ANOVA with Tukey–Kramer post hoc test.

## Supplementary Information

Supplementary Information 1.

Supplementary Information 2.

Supplementary Information 3.

## Data Availability

All the raw data of western blot analysis and bioinformatics analysis are available as supplementary data. Any other data generated or analyzed during this study are available from the corresponding authors on reasonable request. Publically available data provided from the Cancer Genome Atlas Research Network have been downloaded from the cBioPortal site (https://cbioportal-datahub.s3.amazonaws.com/lihc_tcga.tar.gz). Publically available data provided from the Cancer Cell Line Encyclopedia have been downloaded from the DepMap site (https://depmap.org/portal/partials/entity_summary/download?entity_id=21123&dep_enum_name=expression&size_biom_enum_name=none&color=none).
