# Supplementary figures and images for: Loss of SFXN1 mitigates lipotoxicity and predicts poor outcome in non-viral hepatocellular carcinoma
Source: Sci Rep. 2023 Jun 9;13:9449. doi: 10.1038/s41598-023-36660-w (PMC10256799; doi:10.1038/s41598-023-36660-w)

Fig. 3B

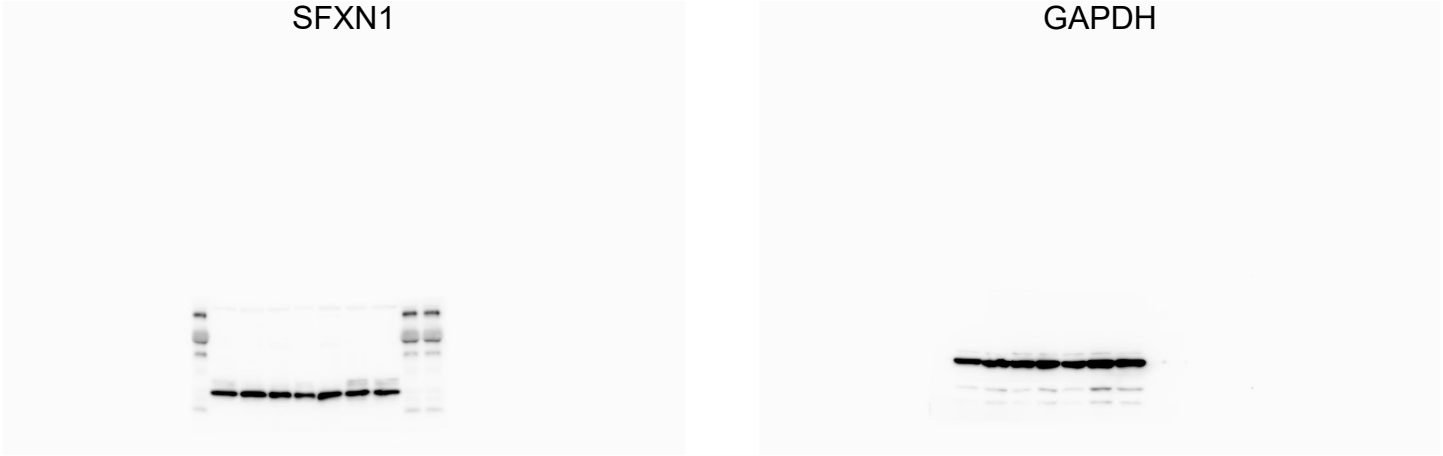

Fig. 3C

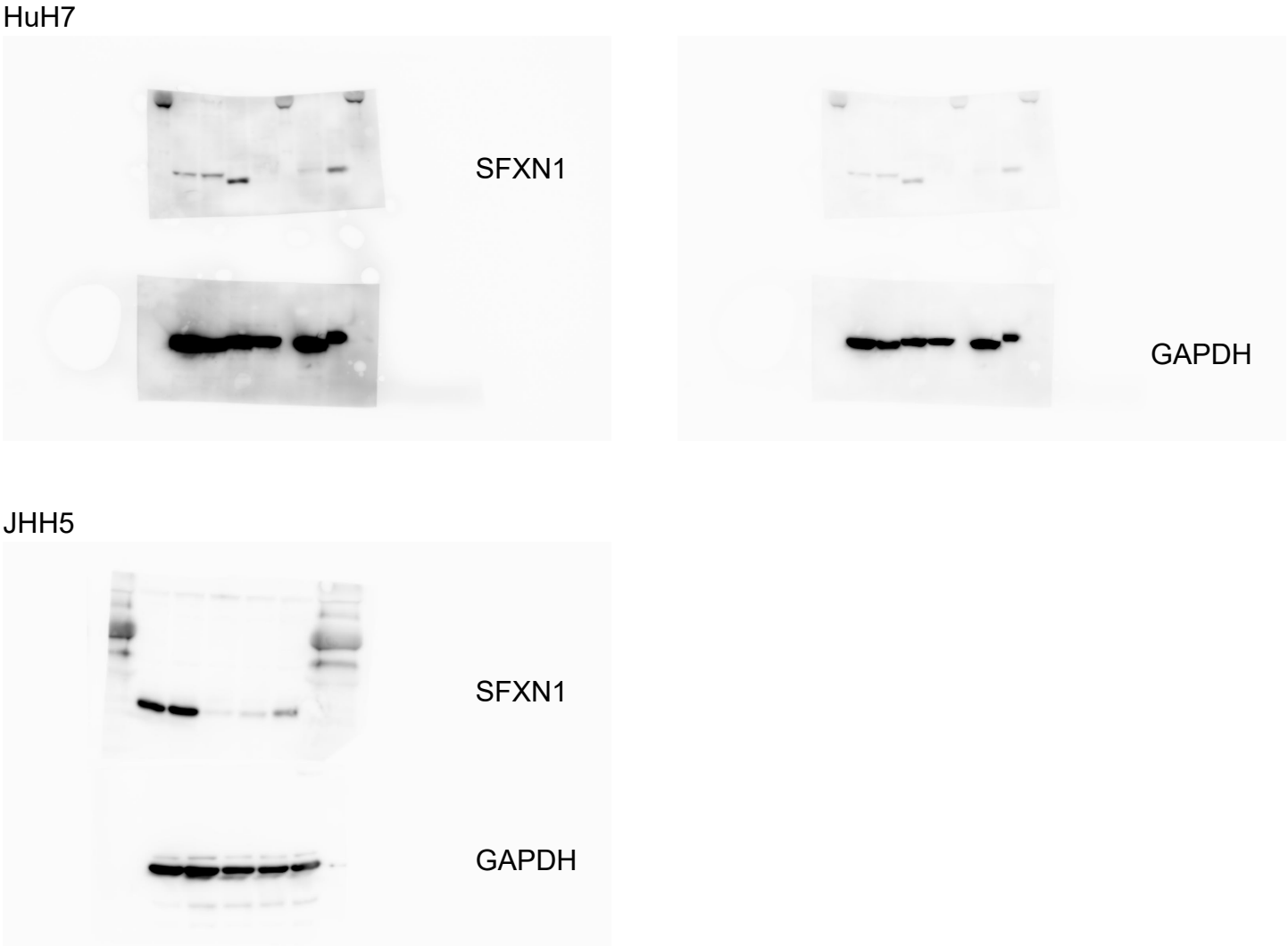

Supplement: Supplementary file 2 — Supplementary Information 2. [file 41598_2023_36660_MOESM2_ESM.pdf]
