# Supplementary material for: Loss of SFXN1 mitigates lipotoxicity and predicts poor outcome in non-viral hepatocellular carcinoma
Source: Sci Rep. 2023 Jun 9;13:9449. doi: 10.1038/s41598-023-36660-w (PMC10256799; doi:10.1038/s41598-023-36660-w)
Supplement: Supplementary file 3 — Supplementary Information 3. [file 41598_2023_36660_MOESM3_ESM.pdf]

Loss of SFXN1 mitigates lipotoxicity and predicts poor outcome in non-viral hepatocellular carcinoma

Kohei Yagi[1,2], Shu Shimada[1,*], Yoshimitsu Akiyama[1], Megumi Hatano[1], Daisuke Asano[2], Yoshiya Ishikawa[2], Hiroki Ueda[2], Shuichi Watanabe[2], Keiichi Akahoshi[2], Hiroaki Ono[2], Minoru Tanabe[2], Shinji Tanaka[1,2,*]

[1]Department of Molecular Oncology, Graduate School of Medicine, Tokyo Medical and Dental University, Tokyo, Japan

[2]Department of Hepato-Biliary-Pancreatic Surgery, Graduate School of Medicine, Tokyo Medical and Dental University, Tokyo, Japan

Corresponding authors

Shu Shimada, M.D., Ph.D.

Department of Molecular Oncology, Graduate School of Medicine, Tokyo Medical and Dental University, 1-5-45 Yushima, Bunkyo-ku, Tokyo 113-8519, Japan

Phone: +81-3-5803-5183; Fax: +81-3-5803-0125; E-mail: shimada.monc@tmd.ac.jp

Shinji Tanaka, M.D., Ph.D., F.A.C.S.

Department of Molecular Oncology, Graduate School of Medicine, Tokyo Medical and Dental University, 1-5-45 Yushima, Bunkyo-ku, Tokyo 113-8519, Japan

Phone: +81-3-5803-5182; Fax: +81-3-5803-0125; E-mail: tanaka.monc@tmd.ac.jp

Supplementary figure legends

Supplementary Figure 1. Evaluation of the intensity score of SFXN1 staining in HCC tissues and adjacent liver tissues. *P*-values are calculated by paired *t*-test. N: adjacent liver tissue; T: tumor tissue.

Supplementary Figure 2. Evaluation of tumor growth of HCC cells under HFD feeding. Tumorigenicity assay of the HuH7-Control cells and the HuH7-SFXN1-KO cells in immunodeficient mice fed with HFD. *P*-values are calculated by Welch's *t*-test. Data are the mean ± SE. HFD: high-fat diet; NS: not significant.

Supplementary Figure 3. Immunohistochemical analysis of tumor tissues derived from the SFXN1-KO HCC cells in immunodeficient mice with HFD. Arrowheads indicate cleaved caspase 3-positive cells. ND: normal diet; HFD: high-fat diet.

Supplementary Fig. 1

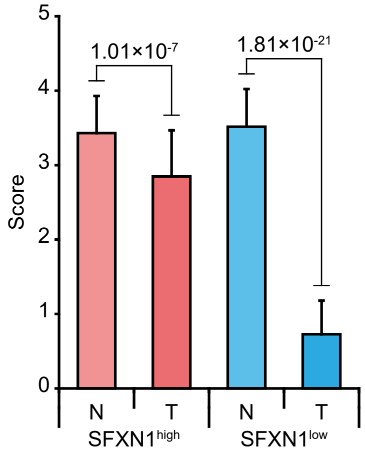

Supplementary Fig. 2

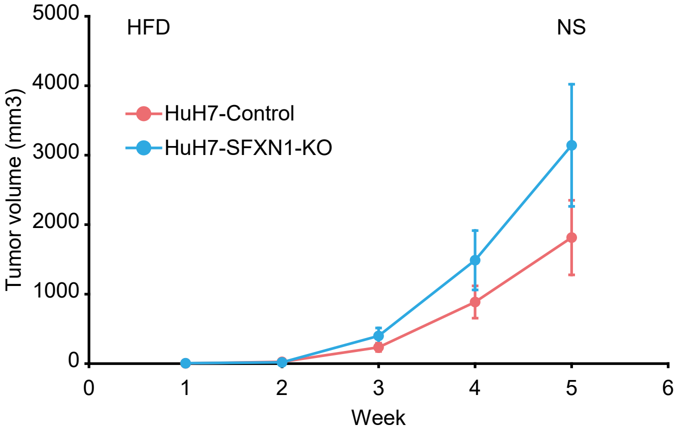

Supplementary Fig. 3

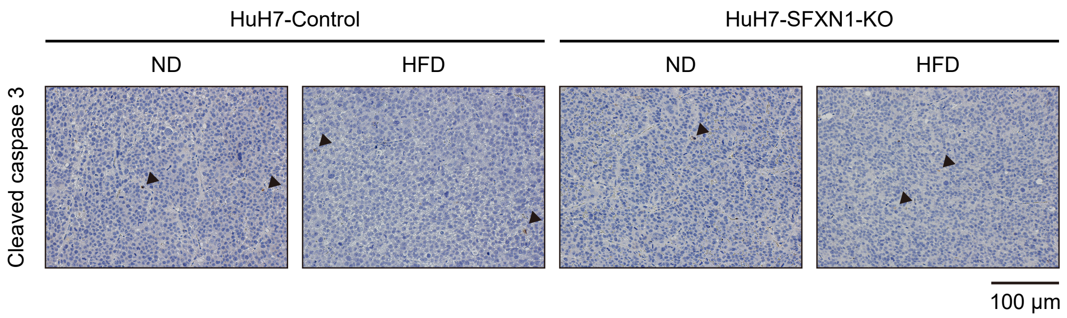

Supplementary Table 1. TOP500 genes.

Gene symbol	Gene ID	*n*	$P_{non\text{-}viral}$	P_{viral}	$\Delta \log P$
BTNL9	153579	92	1.31E-09	0.92	8.85
ADRA1B	147	97	1.69E-09	0.37	8.34
ENG	2022	96	4.47E-09	0.91	8.31
SIK2	23235	180	5.12E-09	0.65	8.10
HYKPP	6329	105	3.33E-08	0.99	7.47
SRL	6345	215	3.81E-09	0.09	7.40
C13orf15	28984	196	3.36E-08	0.80	7.38
ANKRD24	170961	99	2.98E-09	0.04	7.16
TMEM88	92162	126	6.03E-08	0.67	7.05
NMRK1	54981	108	9.93E-08	0.95	6.98
PGM5	5239	112	9.71E-08	0.65	6.82
RAPGEF3	10411	94	9.82E-08	0.61	6.79
ASPA	443	211	1.60E-07	0.75	6.67
MARC2	54996	93	5.11E-08	0.23	6.66
DEXI	28955	92	1.72E-07	0.59	6.54
FAM110D	79927	111	2.79E-07	0.80	6.46
FGB	2244	193	3.00E-07	0.83	6.44
C3orf73	79825	142	2.05E-07	0.55	6.43
CYB5D2	124936	92	3.42E-07	0.74	6.33
CLEC3B	7123	93	4.89E-08	0.10	6.29
RPS27L	51065	99	1.04E-07	0.15	6.16
USHBP1	83878	129	6.51E-07	0.89	6.14
ODF3L2	284451	106	7.41E-07	0.89	6.08
PPAP2A	8611	149	4.03E-07	0.47	6.07
SLC25A25	114789	151	7.19E-07	0.83	6.06
PPARGC1A	10891	190	3.59E-07	0.41	6.06
GPT	2875	123	8.77E-07	0.99	6.05
MMAA	166785	217	8.85E-07	0.90	6.01
VAMP2	6844	100	7.43E-07	0.74	6.00
SHPK	23729	132	8.87E-07	0.86	5.99
ADCY4	196883	92	9.10E-07	0.87	5.98
PXE	368	184	4.59E-07	0.42	5.96
AVPR2	554	111	7.37E-07	0.65	5.95
IREB1	48	128	7.38E-07	0.60	5.91
OXNAD1	92106	126	1.39E-06	1.00	5.86
BAIAP2	10458	110	7.79E-07	0.55	5.85
FLJ31318	375775	208	1.38E-06	0.98	5.85
EXOC3L2	90332	169	5.80E-07	0.39	5.83
LOC100996774	256949	102	6.56E-07	0.37	5.76
G6PC	2538	163	1.56E-06	0.85	5.74
CD300LG	146894	134	1.82E-06	0.94	5.71

DLX6	5166	131	1.74E-06	0.89	5.71
KAZN	23254	124	1.45E-06	0.71	5.69
LOC119865	5138	121	1.59E-06	0.76	5.68
LOC115919	922	144	1.62E-06	0.74	5.66
TMEM204	79652	140	6.00E-07	0.27	5.65
PRSS36	146547	94	2.36E-06	0.97	5.61
PER1	5187	136	2.43E-06	0.87	5.56
FLJ36113	140699	100	1.07E-06	0.37	5.54
HPX	3263	136	2.16E-06	0.61	5.45
MEIS3P1	4213	93	3.54E-06	0.97	5.44
LOC137671	10247	127	2.03E-06	0.55	5.43
DNASE1L3	1776	250	1.34E-06	0.32	5.38
CFHR4	10877	172	4.47E-07	0.11	5.38
SLC45A1	50651	93	3.60E-06	0.80	5.35
GIMAP5	55340	128	4.33E-06	0.95	5.34
GABARAPL1	23710	110	1.51E-06	0.30	5.30
GADD45G	10912	167	2.85E-06	0.55	5.29
ESR1	2099	170	1.36E-06	0.25	5.27
LOC115706	98	129	3.10E-06	0.56	5.26
CLEC16A	23274	105	2.73E-06	0.49	5.25
SYBU	55638	135	4.36E-06	0.76	5.24
PTGER3	5733	112	2.55E-06	0.44	5.23
SORD	6652	129	3.69E-06	0.62	5.22
ACSM2A	123876	112	2.17E-06	0.36	5.22
ADRA2B	151	126	2.92E-06	0.45	5.19
ECHDC2	55268	164	5.08E-06	0.78	5.18
ANGPTL1	9068	99	6.22E-06	0.95	5.18
C8ORF46	254778	126	4.98E-06	0.75	5.18
RAMP3	10268	93	2.91E-06	0.43	5.17
SOCS2	8835	146	8.26E-08	0.01	5.16
PLVAP	83483	152	6.34E-06	0.86	5.13
LYRM5	144363	153	7.02E-06	0.94	5.13
LGSN	51557	102	7.34E-06	0.96	5.12
GNA14	9630	244	1.58E-06	0.20	5.10
LOC82154	2949	137	7.96E-06	0.97	5.09
HADH	3033	197	8.26E-06	0.98	5.08
ECM2	1842	98	5.39E-06	0.61	5.05
NDFIP1	80762	104	7.58E-06	0.78	5.01
SORBS2	8470	112	9.10E-07	0.09	5.01
CRY2	1408	196	6.51E-06	0.65	5.00
TRIM69	140691	125	6.32E-06	0.59	4.97
MAN2C1	4123	93	4.36E-07	0.04	4.95
LOC100133260	84163	113	4.53E-06	0.40	4.95

SLC2A4	6517	95	1.05E-05	0.93	4.95
RGS6	9628	129	1.14E-05	1.00	4.94
COL9A1	1297	137	1.03E-05	0.90	4.94
CCL14	6358	158	9.81E-07	0.08	4.93
NAGLU	4669	95	4.16E-06	0.34	4.92
EMCN	51705	207	4.42E-06	0.36	4.91
DUSP1	1843	92	3.79E-06	0.31	4.91
TMEM100	55273	96	8.80E-06	0.70	4.90
GIMAP1	170575	141	2.58E-06	0.20	4.89
KFSD	6303	125	1.01E-05	0.77	4.88
ROBO4	54538	129	8.68E-06	0.64	4.87
SLC25A34	284723	97	1.43E-05	0.98	4.83
LMOD1	25802	95	1.19E-05	0.79	4.82
TMEM192	201931	160	7.59E-06	0.50	4.82
LPRS2	135138	142	1.19E-05	0.74	4.79
BAAT	570	101	1.70E-05	0.99	4.77
STARD5	80765	171	7.51E-06	0.43	4.75
GALNT15	117248	193	1.16E-05	0.66	4.75
SELP	6403	96	3.18E-06	0.18	4.75
LOC115245	84734	177	1.79E-05	0.98	4.74
TIE1	7075	104	1.57E-05	0.85	4.73
DRD1	1812	224	1.63E-05	0.85	4.72
C6ORF201	404220	122	1.58E-05	0.81	4.71
FABP4	2167	102	1.90E-05	0.95	4.70
GLYD	9380	106	1.93E-05	0.94	4.69
LINC01554	202299	115	2.06E-05	0.99	4.68
FAM122A	116224	211	1.90E-05	0.87	4.66
CYP2C8	1558	121	2.11E-05	0.97	4.66
CCM2L	140706	160	1.81E-05	0.82	4.65
HHH	10166	194	3.31E-06	0.14	4.63
GPT2	84706	209	2.18E-05	0.93	4.63
RGMA	56963	125	8.77E-06	0.37	4.63
TPSG1	25823	93	5.89E-06	0.25	4.62
TAPT1	202018	136	5.70E-06	0.24	4.62
LOC221402	10231	127	1.79E-05	0.75	4.62
SH3GBR	6450	95	2.17E-05	0.86	4.60
CYP3A5	1577	239	1.05E-05	0.41	4.59
VMCM	7010	124	1.84E-05	0.71	4.59
PDE7B	27115	183	1.83E-05	0.68	4.57
SPNS2	124976	99	2.68E-05	1.00	4.57
ZNF276	92822	184	2.51E-05	0.92	4.57
C1R	715	107	1.55E-05	0.55	4.55
PSD4	23550	99	7.31E-08	0.00	4.54

LINC00261	140828	226	6.00E-06	0.20	4.53
DMRV	10020	141	3.04E-05	0.99	4.51
KLF2	10365	111	5.23E-06	0.17	4.51
LOC102724516	7763	224	1.10E-05	0.35	4.51
FGA	2243	213	2.68E-05	0.85	4.50
LOC86701	8991	188	2.81E-05	0.89	4.50
FLT4	2324	103	2.02E-05	0.64	4.50
CDC14B	8555	248	2.56E-05	0.80	4.49
PHLDA3	23612	117	2.67E-05	0.82	4.49
LOC100507145	399959	241	2.35E-05	0.70	4.47
SDPR	8436	138	1.21E-05	0.36	4.47
FGGY	55277	112	3.46E-05	0.99	4.46
PLCB4	5332	105	2.19E-05	0.61	4.44
AASS	10157	152	1.57E-05	0.44	4.44
RABEP1	9135	197	3.10E-05	0.86	4.44
LINGO4	339398	110	2.91E-05	0.80	4.44
ZCCHC24	219654	197	2.16E-05	0.57	4.42
CYB5D1	124637	225	3.65E-05	0.96	4.42
NLRP6	171389	96	3.44E-05	0.88	4.41
CKMT2	1160	122	3.65E-05	0.93	4.41
CDC37L1	55664	147	3.73E-05	0.90	4.38
FGG	2266	175	3.32E-05	0.79	4.38
BTN3A3	10384	152	3.79E-05	0.89	4.37
ZNF49	339327	233	3.14E-05	0.73	4.37
MAOB	4129	180	1.26E-05	0.29	4.37
NPR1	4881	132	1.07E-05	0.25	4.37
LOC122654	6038	103	3.92E-06	0.09	4.36
CYP11A1	1583	135	1.46E-05	0.33	4.35
KIAA0679	10724	184	2.06E-05	0.46	4.35
APOLD1	81575	170	4.22E-05	0.94	4.35
FCN3	8547	95	3.94E-05	0.84	4.33
IL24	11009	234	4.58E-05	0.97	4.33
LOC92033	27068	159	2.67E-05	0.55	4.32
CASP12	120329	222	2.01E-05	0.41	4.31
PEX11G	92960	106	1.01E-05	0.21	4.31
ETNPPL	64850	98	4.32E-05	0.87	4.30
TMEM143	55260	158	3.71E-05	0.73	4.30
CASQ2	845	113	2.26E-05	0.44	4.29
FAM20A	54757	98	3.20E-05	0.61	4.28
CBX7	23492	106	4.66E-05	0.89	4.28
LOC130203	1373	204	1.24E-05	0.24	4.28
MCCC2	64087	234	2.98E-05	0.54	4.26
HSPC057	1540	101	4.11E-05	0.74	4.25

JAM2	58494	94	3.75E-05	0.67	4.25
LOC102724905	9962	210	3.03E-05	0.54	4.25
COQ4	51117	161	5.58E-05	1.00	4.25
RPS6KA2	6196	233	5.70E-05	1.00	4.24
SLC16A11	162515	93	5.80E-05	0.99	4.23
LOC642940	339483	100	5.90E-05	1.00	4.23
ASPRV1	151516	157	5.19E-05	0.87	4.22
SCN4B	6330	120	5.67E-05	0.93	4.22
LINC00324	284029	198	5.62E-06	0.09	4.21
ALAD	210	229	2.83E-05	0.45	4.20
POU6F1	5463	231	5.37E-05	0.85	4.20
COL25A1	84570	132	1.97E-05	0.31	4.20
SEC31B	25956	217	5.78E-05	0.90	4.19
NDRG2	57447	180	7.32E-06	0.11	4.17
SLC46A3	283537	119	6.23E-05	0.91	4.16
ETS2	2114	138	3.84E-05	0.56	4.16
C8A	731	176	6.80E-05	0.97	4.15
EXOC3L1	283849	95	6.62E-05	0.93	4.15
RUNX1-IT1	80215	196	3.71E-05	0.51	4.14
GJA4	2701	170	3.03E-05	0.40	4.12
GFRA2	2675	163	5.09E-05	0.66	4.11
HPN-AS1	100128675	134	6.02E-05	0.78	4.11
ANX14	11199	215	7.68E-06	0.10	4.11
METTL20	254013	145	5.28E-05	0.67	4.10
TMEM217	221468	152	1.92E-05	0.24	4.09
DNM3	26052	92	1.67E-05	0.20	4.08
FAM214A	56204	207	2.08E-05	0.25	4.08
GNG11	2791	125	8.13E-05	0.98	4.08
GFOD1	54438	214	6.70E-05	0.79	4.07
ACSM2B	348158	100	7.22E-05	0.84	4.06
ECSCR	641700	119	7.64E-05	0.85	4.05
BTD	686	92	2.82E-05	0.31	4.03
DKFZp761K1423	23362	128	9.14E-05	0.98	4.03
LINC00426	100188949	93	6.04E-05	0.65	4.03
ALPL	249	93	4.60E-05	0.49	4.03
LRRC32	2615	127	8.37E-05	0.89	4.03
P2RY8	286530	111	9.58E-05	1.00	4.02
CCDC42	146849	119	4.68E-05	0.49	4.02
APOC1	341	94	4.80E-06	0.05	4.02
CRX	1406	134	8.31E-05	0.86	4.02
MGC5363	5583	98	8.74E-05	0.91	4.02
CHAD	1101	92	5.65E-05	0.57	4.00
ACAT1	38	109	3.23E-05	0.32	3.99

DKK3	27122	145	5.11E-05	0.49	3.98
CPEB3	22849	103	9.61E-05	0.92	3.98
PROK1	84432	121	3.38E-05	0.32	3.98
RTN4RL1	146760	92	5.46E-05	0.52	3.98
TMEM187	8269	92	8.42E-05	0.78	3.97
C8B	732	214	1.83E-05	0.17	3.96
ADGRF5	221395	98	4.89E-05	0.44	3.95
ETFDH	2110	137	2.93E-05	0.26	3.95
ZMAT1	84460	139	1.09E-04	0.94	3.93
PITPNM3	83394	139	7.15E-05	0.60	3.93
NKAPL	222698	180	1.16E-04	0.97	3.92
FLJ45737	23145	120	6.40E-05	0.53	3.92
CTH	1491	146	9.76E-05	0.81	3.92
ATG4A	115201	122	1.17E-04	0.97	3.92
CYP4V2	285440	98	1.28E-05	0.11	3.92
SPATA18	132671	101	7.38E-05	0.60	3.91
LOC145786	283659	98	6.10E-05	0.49	3.91
SHROOM4	57477	103	1.21E-04	0.97	3.90
LCAT	3931	149	1.40E-06	0.01	3.89
HAAO	23498	97	1.11E-04	0.86	3.89
NUDT6	11162	250	3.68E-05	0.29	3.89
C7	730	141	5.83E-05	0.44	3.88
FITM1	161247	181	7.91E-05	0.59	3.87
SPARCL1	8404	134	1.33E-04	0.98	3.87
SLCO2A1	6578	98	1.08E-04	0.77	3.85
LDB2	9079	92	5.12E-05	0.36	3.85
PAMR1	25891	204	1.26E-04	0.86	3.83
DEFB132	400830	143	1.07E-04	0.71	3.82
COLEC10	10584	212	3.09E-05	0.20	3.82
GARNL3	84253	227	5.66E-05	0.37	3.81
CACNA2D2	9254	137	6.46E-05	0.42	3.81
RPL34-AS1	285456	259	7.25E-05	0.46	3.81
NFIX	4784	143	1.15E-04	0.73	3.81
LIPE	3991	124	1.12E-04	0.70	3.80
ADI1	55256	118	1.39E-04	0.87	3.80
N4BP2L1	90634	258	7.09E-05	0.44	3.80
AKU	412	167	7.14E-05	0.43	3.78
NAA38	84316	132	6.27E-05	0.38	3.78
C14ORF159	80017	93	1.49E-04	0.89	3.77
F11	2160	150	1.88E-05	0.11	3.77
SSTR1	6751	92	1.28E-04	0.76	3.77
REEP5	7905	109	1.31E-04	0.77	3.77
SUGT1P3	283507	132	5.98E-05	0.35	3.77

LOC101928690	5519	209	9.61E-05	0.56	3.76
SYNPO	11346	96	1.69E-04	0.97	3.76
PAIP2B	400961	193	1.61E-04	0.93	3.76
FOXO1	2308	258	1.12E-04	0.64	3.76
ADH4	127	259	2.27E-05	0.13	3.76
EPM2A	7957	127	1.69E-04	0.96	3.75
LOC101928757	4360	126	1.29E-04	0.71	3.74
SNF1LK	150094	142	1.55E-04	0.85	3.74
CYP2U1	113612	169	1.64E-04	0.89	3.73
COL15A1	1306	116	1.39E-04	0.74	3.73
RANBP10	57610	102	7.98E-05	0.42	3.72
WDR81	124997	119	1.14E-04	0.60	3.72
HMGCS2	3158	163	1.65E-05	0.09	3.72
DFNB44	107	103	1.23E-04	0.64	3.72
STX1B	112755	218	1.88E-04	0.97	3.71
PTPRB	5787	124	1.74E-04	0.89	3.71
DKFZP566B133	4616	97	5.54E-05	0.28	3.71
LOC82212	1548	97	1.43E-04	0.71	3.70
ACADM	34	173	1.99E-04	0.99	3.70
GILZ	1831	102	1.02E-04	0.50	3.69
C3ORF36	80111	122	1.69E-04	0.81	3.68
LILRB5	10990	94	1.62E-04	0.76	3.67
ACY3	91703	106	1.58E-04	0.74	3.67
C4BPA	722	192	1.40E-04	0.65	3.67
LOC56966	56965	92	2.12E-04	0.98	3.66
ASTN1	460	96	9.09E-05	0.42	3.66
ECI1	1632	106	2.99E-05	0.14	3.66
NOSTRIN	115677	124	2.16E-04	0.98	3.66
KDM8	79831	266	2.14E-04	0.96	3.65
ODF3B	440836	155	2.14E-04	0.94	3.64
KLF9	687	98	2.07E-04	0.91	3.64
COX4I2	84701	145	1.63E-04	0.71	3.64
SLC9A3R2	9351	127	2.25E-04	0.98	3.64
LAMB2	3913	96	2.16E-04	0.93	3.63
RANBP3L	202151	109	2.21E-04	0.94	3.63
LPIN2	9663	133	1.30E-04	0.55	3.63
PPP1R32	220004	100	6.55E-05	0.28	3.63
METTL14	57721	224	1.75E-04	0.74	3.62
UBE2QL1	134111	118	2.36E-04	0.98	3.62
LOC90246	90246	172	2.35E-04	0.97	3.62
SLC25A30	253512	232	2.12E-04	0.87	3.61
INCA1	388324	99	1.93E-04	0.79	3.61
GK3P	2713	115	1.54E-04	0.62	3.60

LOC100506112	57494	135	2.21E-04	0.88	3.60
RAMP2	10266	97	1.45E-04	0.58	3.60
KIAA0922	23240	122	2.30E-04	0.91	3.60
LOC138369	229	168	6.69E-05	0.26	3.60
CFB	629	104	1.22E-04	0.47	3.59
SMARCA2	6595	171	2.45E-04	0.93	3.58
CSAD	51380	174	8.13E-05	0.31	3.58
MIR99AHG	388815	245	1.01E-04	0.38	3.58
VWF	7450	133	2.27E-04	0.86	3.58
DLL1	28514	254	1.64E-04	0.60	3.57
GPR182	11318	147	8.74E-05	0.32	3.56
MMRN2	79812	96	2.40E-04	0.87	3.56
GPIHBP1	338328	130	2.58E-04	0.94	3.56
DIO3OS	64150	95	2.40E-04	0.86	3.55
TCF25	22980	98	2.66E-04	0.94	3.55
CNDP1	84735	100	1.95E-04	0.67	3.54
SEC14L3	266629	175	1.34E-04	0.46	3.54
ECI2	10455	212	2.21E-04	0.75	3.53
STOM	2040	165	2.53E-04	0.86	3.53
GOLGA1	2800	196	1.47E-04	0.49	3.52
ADH6	130	252	2.59E-04	0.86	3.52
TOB1	10140	172	1.72E-04	0.56	3.52
ADAD2	161931	225	2.90E-04	0.95	3.52
DNMBP-AS1	100188954	130	1.36E-04	0.44	3.51
C8G	733	92	1.93E-04	0.63	3.51
XAF1	54739	118	1.01E-04	0.33	3.51
LOC221563	2550	92	2.79E-04	0.90	3.51
RPAIN	84268	164	1.19E-04	0.38	3.51
HIF3A	64344	105	1.98E-04	0.64	3.51
CIDEB	27141	125	1.67E-04	0.54	3.51
PPP1R12B	4660	256	1.72E-04	0.55	3.50
ERMARD	55780	271	3.04E-04	0.97	3.50
PDCD1LG2	80380	101	2.33E-04	0.74	3.50
SNX1	6642	189	1.44E-04	0.45	3.50
LOC147680	1	246	1.18E-04	0.37	3.50
NPY1R	4886	132	2.61E-04	0.81	3.49
NCOR1	9611	216	2.28E-04	0.71	3.49
PIK3R1	5295	125	3.12E-04	0.96	3.49
CLEC14A	161198	133	2.78E-04	0.85	3.48
TAFI95	9013	102	1.60E-04	0.48	3.48
PATL2	197135	177	2.24E-04	0.68	3.48
CBFA2T3	863	105	5.63E-05	0.17	3.48
SERTAD1	29950	94	2.57E-04	0.77	3.48

TPPP2	122664	173	5.62E-05	0.17	3.47
CD244	51744	115	2.55E-04	0.76	3.47
ANKFY1	51479	263	3.04E-04	0.90	3.47
ADRB2	154	92	1.79E-04	0.53	3.47
CO	358	103	2.14E-04	0.63	3.47
HCG26	352961	115	2.73E-04	0.80	3.47
DERA	51071	154	2.94E-04	0.86	3.47
LOC95514	28968	274	2.15E-04	0.63	3.47
FLJ25459	246175	252	3.20E-04	0.93	3.46
CPS1-IT1	29034	105	8.50E-05	0.25	3.46
DHODH	1723	118	2.21E-04	0.63	3.45
UBR3	130507	205	3.45E-04	0.98	3.45
TSLC1	23705	106	2.75E-04	0.78	3.45
RABGGTA	5875	119	3.52E-04	0.99	3.45
IRAG	10335	92	2.18E-04	0.61	3.45
LOC115149	3075	106	1.26E-04	0.35	3.44
PDGFD	80310	220	2.52E-04	0.69	3.44
IGF1	3479	115	3.43E-04	0.94	3.44
RPL21P44	402176	116	3.61E-04	0.99	3.44
FAS	355	275	3.00E-04	0.81	3.43
IL1RL1	9173	223	1.57E-04	0.42	3.43
PROZ	8858	168	1.24E-04	0.33	3.42
PRKAG2	51422	95	2.66E-04	0.70	3.42
CYP3A43	64816	156	2.78E-04	0.73	3.42
TAT	6898	98	2.46E-04	0.65	3.42
FHL5	9457	105	3.55E-04	0.93	3.42
C16ORF71	146562	93	2.88E-04	0.75	3.42
ADPRM	56985	104	1.55E-04	0.40	3.42
PTK2B	2185	223	9.31E-05	0.24	3.41
ADGRE1	2015	164	3.30E-04	0.85	3.41
FLJ10530	60528	157	3.18E-04	0.81	3.41
GIMAP8	155038	188	8.92E-05	0.23	3.41
MASP2	10747	130	2.66E-04	0.68	3.41
PALM2	114299	93	1.66E-04	0.42	3.41
LOC197414	3681	96	3.28E-04	0.83	3.40
MCEE	84693	92	2.70E-04	0.68	3.40
ZSCAN1	284312	129	2.92E-04	0.73	3.40
CBLN1	869	163	3.94E-04	0.98	3.40
PRICKLE1	144165	97	6.16E-05	0.15	3.39
LOC94533	10171	238	2.41E-04	0.59	3.39
PPAP2B	8613	229	7.80E-05	0.19	3.39
POR	5447	138	3.44E-04	0.83	3.38
ASCL1	429	192	4.00E-04	0.97	3.38

LOC115824	2840	111	2.25E-04	0.54	3.38
APOL6	80830	94	2.04E-04	0.49	3.38
CFI	3426	162	2.79E-04	0.66	3.37
PNPLA4	8228	114	4.19E-04	0.99	3.37
LOC199828	947	202	3.30E-04	0.76	3.36
KLRC4	8302	98	2.98E-04	0.69	3.36
FTX	100302692	146	3.45E-04	0.80	3.36
IGIP	492311	92	2.62E-04	0.60	3.36
EPD	501	134	3.25E-04	0.73	3.35
DBT	1629	190	1.32E-04	0.30	3.35
GYS2	2998	191	6.10E-05	0.14	3.35
BOC	91653	191	1.66E-04	0.36	3.34
EPB41L4A-AS2	54508	94	3.48E-04	0.76	3.34
FBXL5	26234	93	5.93E-05	0.13	3.34
KBTBD11	9920	206	6.83E-05	0.15	3.34
CYP4F3	4051	217	3.71E-04	0.80	3.33
CXCL2	2920	158	4.08E-04	0.88	3.33
INS-IGF2	723961	270	3.98E-04	0.84	3.33
AKAP6	9472	97	1.05E-04	0.22	3.33
FBXO8	26269	135	2.97E-04	0.62	3.32
MRT2A	51185	275	2.78E-04	0.57	3.32
C9orf130	100128782	104	4.60E-04	0.95	3.32
MAMDC2	256691	92	1.33E-04	0.27	3.31
TCF21	6943	164	5.72E-05	0.12	3.31
ADHFE1	137872	270	3.45E-04	0.71	3.31
ANGPT4	51378	103	4.22E-04	0.86	3.31
CCDC85A	114800	116	1.94E-04	0.39	3.30
CTSO	1519	98	3.38E-04	0.67	3.30
MTMR10	54893	184	4.57E-04	0.90	3.30
PKD1L3	342372	267	2.04E-04	0.40	3.30
SIDT2	51092	140	4.02E-04	0.78	3.29
WFDC1	58189	198	5.11E-04	0.99	3.29
C15ORF43	145645	267	4.54E-04	0.88	3.29
TAL1	6886	96	3.73E-04	0.72	3.28
ALDH2	217	129	2.21E-04	0.42	3.28
FXYD6	53826	147	6.29E-05	0.12	3.28
AKT2	208	176	2.16E-04	0.41	3.28
HIGD1A	25994	111	4.62E-04	0.88	3.28
LOC116417	9344	96	2.34E-04	0.44	3.28
LILRB2	10288	132	3.93E-04	0.75	3.28
LOC94378	5098	176	2.28E-04	0.43	3.27
MUT	4594	188	1.49E-04	0.28	3.27
ATF7IP2	80063	106	7.02E-06	0.01	3.27

OVD1A	593	267	4.65E-04	0.86	3.27
LRRC55	219527	103	3.47E-04	0.64	3.27
PCDHB4	56131	97	4.96E-04	0.92	3.27
MBL1P	8512	165	4.30E-04	0.79	3.26
CDKN1A	1026	95	5.09E-04	0.94	3.26
AIF1L	83543	100	4.36E-04	0.80	3.26
TLR3	7098	158	5.24E-04	0.95	3.26
P2RY13	53829	110	2.28E-04	0.41	3.26
PGN	6687	108	5.38E-04	0.96	3.25
SLC25A28	81894	105	5.27E-04	0.94	3.25
C3P1	388503	203	3.18E-04	0.56	3.25
C1S	716	102	4.24E-04	0.75	3.25
PHYKPL	85007	94	5.57E-04	0.98	3.24
DMGDH	29958	167	4.48E-05	0.08	3.24
NTF3	4908	124	3.28E-04	0.57	3.24
TMEM220	388335	209	3.46E-05	0.06	3.24
KCNJ8	3764	276	5.63E-04	0.98	3.24
FAM107A	11170	226	4.44E-04	0.77	3.24
DUSP10	11221	206	1.98E-04	0.34	3.24
FLJ23157	54457	109	5.51E-04	0.95	3.24
PON1	5444	205	1.06E-04	0.18	3.24
OGDHL	55753	101	2.23E-04	0.38	3.23
LOC87229	54861	176	3.28E-04	0.56	3.23
HDC	3067	121	1.48E-04	0.25	3.23
UQCRC2	7385	101	5.10E-04	0.87	3.23
LOC92771	57644	177	5.85E-04	0.99	3.23
HP	3240	104	4.84E-04	0.82	3.23
C1QTNF7	114905	132	4.74E-04	0.80	3.23
LOC89993	5924	265	5.85E-04	0.99	3.23
LOC105379131	1036	183	4.79E-04	0.81	3.23
COG3	83548	235	5.03E-04	0.85	3.23
C1QL2	165257	175	1.68E-04	0.28	3.22
SYNPO2	171024	169	1.66E-04	0.27	3.22
BDH1	622	106	1.90E-05	0.03	3.22
DECR1	1666	156	5.62E-04	0.92	3.22
FAM151B	167555	92	3.61E-04	0.59	3.22
ARHGEF9	23229	150	5.93E-04	0.96	3.21
LOC93212	2346	187	3.56E-04	0.58	3.21
PYGM	5837	104	1.21E-04	0.19	3.21
KCNA5	3741	92	4.22E-04	0.68	3.21
HES5	388585	173	5.33E-04	0.85	3.20
A-362G6.1	9665	156	4.96E-04	0.79	3.20
EIF4E3	317649	164	4.86E-04	0.77	3.20

INPP5K	51763	144	4.01E-04	0.63	3.20
ACAD11	84129	174	4.47E-04	0.70	3.19
EDNRB	1910	98	5.19E-04	0.81	3.19
GABARAPL3	23766	266	3.47E-04	0.54	3.19
ZNF181	339318	204	2.95E-04	0.46	3.19
C10ORF105	414152	104	2.25E-04	0.35	3.19
APOA5	116519	235	2.63E-04	0.41	3.19
LMO2	4005	118	2.80E-04	0.43	3.19
NFYB	4801	105	3.41E-04	0.52	3.18
STBD1	8987	136	3.38E-04	0.52	3.18
EBF3	253738	180	3.10E-04	0.47	3.18
DNAJC25	548645	161	4.37E-04	0.67	3.18
MOCOD	4337	121	6.45E-04	0.98	3.18
BTN3A1	11119	224	2.19E-04	0.33	3.18
MLXIPL	51085	174	6.58E-04	1.00	3.18
GALNT16	57452	106	5.04E-04	0.76	3.18
INMT	11185	105	2.68E-05	0.04	3.18
OCEL1	79629	197	3.93E-04	0.58	3.17
MLYCD	23417	184	2.86E-04	0.42	3.17
MCHR1	2847	240	3.63E-04	0.53	3.17
NR3C2	4306	238	4.06E-04	0.60	3.17
IVD	3712	219	9.89E-05	0.14	3.17
LOC100131345	285175	230	6.04E-04	0.88	3.17
SFXN1	94081	146	6.82E-04	0.99	3.16
LOC220538	55889	214	2.57E-04	0.37	3.16
RAI2	10742	137	3.49E-04	0.50	3.16
LOC116122	635	122	6.84E-04	0.98	3.16
CYYR1	116159	185	1.55E-04	0.22	3.15
FAM19A1	407738	276	6.11E-04	0.87	3.15

Supplementary Table 2. Annotations enriched in the TOP500 genes.

ID	Term	FDR
GO:0005759	Mitochondrial matrix	2.86E-10
hsa01100	Metabolic pathways	1.20E-08
hsa04610	Complement and coagulation cascades	2.73E-07
KW-0496	Mitochondrion	5.10E-07
hsa00280	Valine, leucine and isoleucine degradation	7.64E-06
KW-0180	Complement pathway	3.14E-05
GO:0072562	Blood microparticle	3.27E-05
KW-0443	Lipid metabolism	9.53E-05
KW-0037	Angiogenesis	9.73E-05
GO:0006956	Complement activation	1.04E-04
KW-0560	Oxidoreductase	1.28E-04
hsa00071	Fatty acid degradation	2.00E-04
KW-0179	Complement alternate pathway	2.40E-04
GO:0001525	Angiogenesis	3.01E-04
KW-0456	Lyase	3.18E-04
KW-0276	Fatty acid metabolism	3.76E-04
GO:0005739	Mitochondrion	6.21E-04

GO: GO term; hsa: KEGG pathway; KW: Uniprot; FDR: false discovery rate.

Supplementary Table 3. Inner mitochondrial membrane-related gene set supplied by HPA.

Gene symbol	Gene ID	*n*	$P_{non\text{-}viral}$	P_{viral}	$\Delta \log P$
IREB1	48	128	7.38E-07	0.60	5.91
HADH	3033	197	8.26E-06	0.98	5.08
AASS	10157	152	1.57E-05	0.44	4.44
MCCC2	64087	234	2.98E-05	0.54	4.26
COQ4	51117	161	5.58E-05	1.00	4.25
ACAT1	38	109	3.23E-05	0.32	3.99
SPATA18	132671	101	7.38E-05	0.60	3.91
HMGCS2	3158	163	1.65E-05	0.09	3.72
ACADM	34	173	1.99E-04	0.99	3.70
ECI1	1632	106	2.99E-05	0.14	3.66
ECI2	10455	212	2.21E-04	0.75	3.53
XAF1	54739	118	1.01E-04	0.33	3.51
DHODH	1723	118	2.21E-04	0.63	3.45
EPD	501	134	3.25E-04	0.73	3.35
DBT	1629	190	1.32E-04	0.30	3.35
HIGD1A	25994	111	4.62E-04	0.88	3.28
DMGDH	29958	167	4.48E-05	0.08	3.24
UQCRC2	7385	101	5.10E-04	0.87	3.23
BDH1	622	106	1.90E-05	0.03	3.22
DECR1	1666	156	5.62E-04	0.92	3.22
IVD	3712	219	9.89E-05	0.14	3.17
SFXN1	94081	146	6.82E-04	0.99	3.16
SDHA	6389	130	7.14E-04	0.89	3.09
ACADVL	37	265	5.17E-04	0.56	3.03
TTC19	54902	175	9.28E-04	0.96	3.01
GCDH	2639	173	3.52E-04	0.36	3.01
LOC147548	10449	193	7.28E-04	0.71	2.99
ACSF3	197322	139	1.68E-04	0.16	2.99
NADK2	133686	204	1.01E-03	0.67	2.82
LOC115046	291	135	1.46E-03	0.96	2.82
NDUFB6	4712	175	4.97E-04	0.30	2.79
FDXR	2232	206	1.52E-03	0.93	2.78
HINT2	84681	115	1.05E-03	0.59	2.75
PARP9	83666	275	1.53E-03	0.71	2.67
LOC138179	64975	93	1.95E-03	0.88	2.65
LOC135860	4967	119	1.05E-03	0.39	2.57
ALAS1	211	227	1.08E-03	0.38	2.55
LOC100506517	4329	116	2.37E-03	0.82	2.54
SCZD4	5625	110	2.36E-03	0.80	2.53
HIBCH	26275	108	3.13E-03	0.98	2.49

LOC112759	10240	98	1.12E-03	0.34	2.49
LOC51770	8803	265	3.23E-03	0.94	2.46
RMDN3	55177	234	2.60E-03	0.73	2.45
GRPEL1	80273	267	2.97E-03	0.82	2.44
HSDL2	84263	141	2.55E-03	0.70	2.44
ECHS1	1892	234	4.06E-03	1.00	2.39
SLC25A23	79085	263	2.04E-03	0.48	2.38
IDH2	3418	186	7.89E-04	0.19	2.37
MRPL40	64976	169	4.33E-03	1.00	2.36
SDS	10993	190	4.36E-03	0.99	2.36
SIRT5	23408	111	3.67E-03	0.78	2.33
FDX1	2230	92	1.84E-03	0.38	2.32
NPD009	18	190	9.01E-04	0.17	2.29
NDUFAF1	51103	275	4.94E-03	0.93	2.28
UQCRQ	27089	182	4.63E-03	0.71	2.18
DCAF8	50717	192	4.90E-03	0.74	2.18
ALKBH7	84266	259	4.62E-03	0.69	2.17
PCK2	5106	181	4.80E-03	0.69	2.16
SLC25A42	284439	184	3.90E-03	0.55	2.15
ACADS	35	111	1.62E-04	0.02	2.14
ALDH5A1	7915	172	4.62E-04	0.06	2.13
SELO	83642	144	7.48E-03	0.98	2.12
GHITM	27069	114	7.87E-03	0.96	2.09
NDUFV1	4723	211	8.95E-03	0.99	2.04
CLPX	10845	140	2.09E-03	0.23	2.03
TACO1	51204	256	8.32E-03	0.89	2.03
TPPP	11076	252	8.71E-03	0.89	2.01
POLG	5428	112	5.36E-03	0.51	1.98
TMEM71	137835	227	6.50E-03	0.60	1.96
IDH3A	3419	153	5.79E-03	0.53	1.96
MPC1	51660	274	1.20E-02	0.96	1.90
SUCLG2	8801	100	7.56E-03	0.59	1.89
NAMSD	9131	130	8.57E-03	0.67	1.89
LOC123541	26589	142	3.94E-03	0.29	1.87
IFIT3	3437	124	3.88E-03	0.27	1.84
OXSM	54995	275	1.24E-02	0.83	1.83
MRPS36	92259	114	1.43E-02	0.94	1.82
SIRT1	23411	271	1.43E-02	0.94	1.82
PDK3	5165	98	1.37E-02	0.87	1.80
LOC101928714	594	252	3.91E-03	0.23	1.78
BCKDK	10295	270	6.00E-03	0.36	1.77
FH	2271	221	1.36E-02	0.80	1.77
DDX28	55794	180	1.53E-02	0.81	1.73

MRPL48	51642	110	1.52E-02	0.78	1.71
ADCK4	79934	107	1.09E-02	0.56	1.71
LACTB	114294	140	2.15E-02	0.98	1.66
PCCA	5095	108	1.83E-02	0.82	1.65
NUBPL	80224	193	9.83E-03	0.41	1.62
RAB11FIP3	9727	97	1.87E-02	0.77	1.62
DKFZp564C246	10367	204	2.37E-02	0.97	1.61
ATP5L2	267020	201	7.18E-03	0.29	1.61
DHTKD1	55526	109	8.79E-03	0.36	1.61
GATM	2628	227	1.61E-02	0.63	1.60
ELK3	2004	267	9.46E-03	0.37	1.59
ETFB	2109	179	2.60E-02	0.91	1.54
HADHB	3032	170	8.69E-03	0.30	1.53
MALSU1	115416	248	2.65E-02	0.90	1.53
NLRX1	79671	226	6.06E-03	0.20	1.52
CPT2	1376	157	1.90E-02	0.59	1.49
FAHD1	81889	134	1.16E-02	0.35	1.48
DNAJA3	9093	126	2.95E-02	0.88	1.47
MRPS22	56945	92	3.41E-02	0.99	1.46
CPT1A	1374	234	3.33E-02	0.96	1.46
AK3	50808	240	3.36E-02	0.95	1.45
ATAD3C	219293	259	2.04E-02	0.56	1.44
PRO1304	4719	250	3.56E-02	0.86	1.38
MARCH5	54708	153	3.54E-02	0.85	1.38
KIAA0824	51585	169	3.92E-02	0.94	1.38
LOC112763	51079	242	4.17E-02	0.98	1.37
MAOA	4128	252	3.52E-02	0.81	1.36
FIS1	51024	92	1.67E-02	0.38	1.36
MIPEP	4285	100	1.29E-02	0.27	1.32
LOC100509698	4731	150	4.63E-02	0.96	1.32
COX4I1	1327	97	4.14E-02	0.85	1.31
L2HGDH	79944	174	4.80E-02	0.99	1.31
OTC	5009	108	3.01E-02	0.62	1.31
SLC25A46	91137	262	2.95E-02	0.60	1.31
LOC131448	29083	131	4.53E-02	0.90	1.30
NAGS	162417	141	3.01E-02	0.57	1.28
HK1	3098	101	5.31E-02	1.00	1.27
UQCRC1	7384	186	3.79E-02	0.69	1.26
MCCC1	56922	260	2.06E-02	0.37	1.25
C10ORF10	11067	111	4.74E-02	0.81	1.23
CMC4	100272147	276	5.88E-02	0.99	1.23
C19ORF70	125988	172	5.04E-02	0.85	1.23
ACSL5	51703	147	5.01E-02	0.81	1.21

ATP5J	522	261	1.34E-02	0.21	1.19
D2HGDH	728294	276	5.96E-02	0.91	1.18
RNMTL1	55178	254	4.52E-02	0.66	1.16
NUDT9	53343	186	4.66E-02	0.67	1.16
KIAA0567	4976	105	2.71E-02	0.39	1.16
IBA57	200205	141	6.33E-02	0.89	1.15
DLST	1743	103	3.58E-02	0.48	1.13
FLJ11328	84196	172	7.36E-02	0.98	1.12
S1PR4	8698	115	1.29E-02	0.17	1.12
LOC654185	36	229	3.27E-02	0.43	1.12
MECR	51102	167	3.28E-02	0.42	1.10
PPM1K	152926	244	4.61E-03	0.06	1.10
FOXRED1	55572	180	7.67E-02	0.95	1.09
CCDC109B	55013	95	7.48E-02	0.91	1.08
LOC91471	3313	102	4.71E-02	0.57	1.08
MDH2	4191	152	7.95E-02	0.94	1.07
UQCRFS1	7386	273	2.71E-02	0.31	1.06
COX5B	1329	276	6.42E-02	0.71	1.05
PLSCR3	57048	123	8.81E-02	0.94	1.03
MRPL39	54148	199	8.20E-02	0.85	1.02
NDUFAB1	4706	186	8.62E-02	0.89	1.01
NDUFS3	4722	121	9.55E-02	0.98	1.01
TMLHE	55217	240	4.84E-02	0.48	1.00
BRP17	25953	157	4.95E-02	0.48	0.98
MRPL28	10573	105	1.01E-01	0.95	0.98
ADCK3	56997	211	1.03E-01	0.97	0.97
SLC25A20	788	184	4.09E-02	0.37	0.96
PDHA1	5160	173	7.35E-02	0.67	0.96
PHB2	11331	270	8.11E-02	0.74	0.96
COX15	1355	97	1.77E-02	0.15	0.94
PCHC	6390	274	6.04E-02	0.53	0.94
NDUFA7	4701	233	5.01E-02	0.41	0.92
SLC25A13	10165	166	4.66E-02	0.37	0.90
TXNRD2	10587	259	1.06E-01	0.84	0.90
COA3	28958	271	1.13E-01	0.89	0.90
DHFRL1	200895	108	2.39E-02	0.19	0.89
LETMD1	25875	94	8.98E-02	0.70	0.89
CCM2	83605	108	9.03E-02	0.69	0.88
GATB	5188	104	2.84E-02	0.21	0.88
LOC115699	23474	156	7.33E-02	0.51	0.84
SDHAF1	644096	130	1.08E-01	0.75	0.84
MRPS18B	28973	171	1.35E-01	0.93	0.84
SLC11A2	4891	269	1.36E-01	0.88	0.81

MRP63	78988	92	1.18E-01	0.72	0.78
AMT	275	174	1.48E-01	0.89	0.78
LOC221701	3096	274	1.55E-01	0.92	0.77
TST	7263	270	1.77E-01	0.98	0.74
FLJ27465	79705	92	1.30E-01	0.71	0.74
NDUFA11	126328	230	1.50E-01	0.79	0.72
GSTP1	2950	168	1.60E-01	0.85	0.72
DHRS2	10202	163	9.74E-02	0.51	0.72
DHFR	1719	225	1.32E-01	0.69	0.72
FASTKD2	22868	170	1.33E-01	0.69	0.72
APOOL	139322	184	1.70E-01	0.88	0.71
KIAA0391	9692	216	1.92E-01	0.97	0.70
CWC15	51503	110	1.36E-01	0.69	0.70
PEMT	10400	227	1.32E-01	0.66	0.70
BCAT2	587	130	1.57E-01	0.78	0.70
ETFA	2108	126	4.06E-02	0.20	0.69
FLJ22512	64963	271	2.05E-01	0.99	0.68
ALDH1B1	219	271	2.55E-02	0.12	0.68
CMPK2	129607	142	1.79E-01	0.83	0.67
ATP5I	521	232	9.03E-02	0.41	0.66
SCA28	10939	214	2.18E-01	1.00	0.66
GLDC	2731	269	1.18E-01	0.53	0.65
MTFR1	9650	123	1.61E-01	0.72	0.65
GCAT	23464	100	2.22E-01	0.97	0.64
LOC101927147	4705	149	2.33E-01	0.97	0.62
SMDT1	91689	225	1.95E-01	0.81	0.62
XPC	7508	162	1.39E-01	0.57	0.62
SLC44A1	23446	191	2.21E-01	0.89	0.61
FAM110B	90362	103	2.10E-01	0.83	0.60
AURKAIP1	54998	132	1.50E-01	0.59	0.59
ATG4D	84971	203	2.23E-01	0.87	0.59
MRPL15	29088	272	2.53E-01	0.97	0.58
PC	5091	139	1.44E-01	0.53	0.56
TXN2	25828	276	1.60E-01	0.54	0.53
SLC25A10	1468	110	1.01E-01	0.33	0.52
BNIP3L	665	178	1.88E-01	0.62	0.52
LOC119829	7351	180	2.99E-01	0.99	0.52
LOC143968	221120	102	2.70E-01	0.89	0.52
OCD1	627	110	2.29E-02	0.07	0.51
NDUFS2	4720	111	3.02E-01	0.98	0.51
CCR7	1236	214	5.62E-02	0.18	0.51
HADHA	3030	185	2.73E-01	0.84	0.49
NFKB1	4790	261	3.19E-01	0.97	0.48

CLPP	8192	126	1.07E-01	0.32	0.48
SHMT2	6472	104	2.95E-01	0.88	0.48
ACSS3	79611	239	3.12E-01	0.93	0.47
HMGN5	79366	172	2.98E-01	0.88	0.47
ATPIF1	93974	122	3.07E-01	0.90	0.47
LOC100506500	5164	242	9.26E-02	0.27	0.46
ABCB8	11194	247	2.25E-01	0.65	0.46
AIFM3	150209	105	3.46E-01	0.94	0.44
MGARP	84709	203	3.18E-01	0.86	0.43
DCPS	28960	94	3.40E-01	0.91	0.43
TIMM10	26519	246	2.53E-01	0.66	0.42
MGC5618	6648	267	6.14E-02	0.15	0.39
COX14	84987	140	3.29E-01	0.78	0.38
AIM2	9447	193	3.85E-01	0.91	0.37
PRKA1	8165	137	1.55E-01	0.34	0.34
MXD1	4084	177	1.99E-01	0.43	0.33
ANKRD37	353322	95	2.04E-01	0.43	0.33
GLRX5	51218	258	4.36E-01	0.92	0.32
GRSF1	2926	202	2.72E-01	0.55	0.31
TOMM70A	9868	146	3.06E-01	0.60	0.29
FAM124B	79843	110	2.68E-01	0.52	0.29
TFB2M	64216	188	4.78E-01	0.91	0.28
POU5F1B	5462	97	5.02E-01	0.95	0.28
GLUL	2752	121	4.98E-01	0.94	0.28
ALDH1L2	160428	126	3.36E-01	0.63	0.27
TSFM	10102	104	5.72E-01	0.97	0.23
NARS2	79731	161	5.23E-01	0.87	0.22
LOC101928789	11262	276	2.72E-01	0.44	0.21
CREBZF	58487	92	6.53E-01	0.98	0.18
FAM73A	374986	212	7.56E-01	0.98	0.11
NDUFC2	4718	272	4.36E-01	0.51	0.06
MARC1	64757	275	1.98E-01	0.22	0.04
MRPL19	9801	261	6.68E-01	0.58	-0.06
ZBTB6	10773	125	3.42E-01	0.29	-0.08
NCBP1	4686	138	3.83E-01	0.25	-0.18
MLS	3052	135	5.74E-01	0.10	-0.78
XPNPEP3	63929	254	1.89E-01	0.02	-0.93

Supplementary Table 4. Inner mitochondrial membrane-related gene set supplied by GO central.

Gene symbol	Gene ID	*n*	$P_{non\text{-}viral}$	P_{viral}	$\Delta\log P$
ETFDH	2110	137	2.93E-05	0.26	3.95
SFXN1	94081	146	6.82E-04	0.99	3.16
COX18	285521	226	1.69E-03	0.46	2.43
GHITM	27069	114	7.87E-03	0.96	2.09
SFXN2	118980	92	1.07E-03	0.09	1.94
MPC1	51660	274	1.20E-02	0.96	1.90
COQ2	27235	242	3.77E-02	0.93	1.39
LOC129811	94097	107	4.25E-02	0.90	1.33
CCDC109B	55013	95	7.48E-02	0.91	1.08
LOC100506556	63933	170	6.54E-02	0.62	0.98
COA3	28958	271	1.13E-01	0.89	0.90
SMDT1	91689	225	1.95E-01	0.81	0.62
COX16	51241	259	2.43E-01	0.98	0.60

Supplementary Table 5. Inner mitochondrial membrane-related gene set supplied by UniProt.

Gene symbol	Gene ID	*n*	$P_{non\text{-}viral}$	P_{viral}	$\Delta\log P$
ETFDH	2110	137	2.93E-05	0.26	3.95
SFXN1	94081	146	6.82E-04	0.99	3.16
COX18	285521	226	1.69E-03	0.46	2.43
SCO1	6341	178	2.59E-03	0.43	2.22
COQ2	27235	242	3.77E-02	0.93	1.39
CCDC109B	55013	95	7.48E-02	0.91	1.08
LOC100506556	63933	170	6.54E-02	0.62	0.98
COA3	28958	271	1.13E-01	0.89	0.90
SMDT1	91689	225	1.95E-01	0.81	0.62
COX16	51241	259	2.43E-01	0.98	0.60
